# Cognitive and socioemotional growth of youth in the digital era

**DOI:** 10.1016/j.lanwpc.2025.101536

**Published:** 2025-03-28

**Authors:** The Lancet Regional Health – Western Pacific

The forthcoming implementation of the social media ban for Australian children younger than 16 years has triggered wide ranging debate concerning the safety, privacy, and freedom of speech of young people as well as obligations of both parents and technology companies. There is growing scepticism regarding whether age restrictions will be effective as children may easily bypass age restrictions. A recent report from Australia's online safety regulator revealed that a staggering 80% of children aged between 8 and 12 years used social media, with the most frequently used being YouTube, TikTok, and Snapchat, further highlighting the significant challenges and unanswered questions surrounding the feasibility and implementation of the ban. In particular, the proposed exemption of YouTube due to its educational purposes has triggered calls for equal application of the law across all social media services. Central to this discussion is the importance of human connection—the emotional bonds and shared experiences that unite individuals. Social media serves as one such medium for fostering these connections. These connections are more important than ever in our increasingly digital world, especially in the wake of the pandemic, ongoing geopolitical issues, and conflicts that can further divide and isolate individuals and communities.

It is widely acknowledged that the positive and negative impacts of social media use extend to social interaction and emotional expression. For example, social media may provide a platform for young Pacific Islanders to connect and support one another, particularly around sensitive topics like sexuality. However, the harms associated with social media, such as bullying and the targeting of children and adolescents by online predators, are major concerns given the developmental stage of young people, which can make them more susceptible to negative influences and harmful interactions. Overall, there is still very little research specifically examining how social media impacts children's cognitive and socioemotional development. This is crucial given research on the negative effects of digital use on brain development in children, particularly the prefrontal cortex, which plays a vital role in higher-level cognitive functions such as decision making, planning, and social behaviour. Among children aged between 18 and 36 months in Taiwan, those who spent more time using touch screen devices experienced greater emotional and behavioural problems, while language development was unaffected. Consistent with this, conduct problems, peer problems, hyperactivity, and difficulties with prosocial behaviour were associated with more than 2 h of screen time among children aged 4 years in China.

Accordingly, while research in this area is growing, significant gaps still remain. Specifically, large-scale longitudinal studies on multidimensional developmental outcomes are urgently needed to better understand the role of social media in physical, cognitive, emotional, social, and behavioural development, and to identify critical time periods of vulnerability and resilience among young people. This is vital in light of the ongoing challenges associated with an increasingly digital world in the post-pandemic era, where digital learning and remote work have become part of the new norm. As such, it is also essential to examine the potential benefits in light of emerging research pointing to specific advantages that digital technology may offer to young people experiencing displacement in the Western Pacific. These individuals are particularly vulnerable due to disrupted social networks and limited access to mental health support in the aftermath of the pandemic. Among children who are refugees living in Malaysia, digital learning and hybrid learning models were associated with academic readiness. This study also raised the importance of socioemotional learning and skills (eg, emotional regulation, empathy, and social interaction), closely connected to cognitive development. While it is understandable that much of the research relies on caregiver reports, examining the lived experiences of young people with social media must be prioritised as much as possible. Research is particularly limited in lower-income and middle-income countries across the Western Pacific, posing a significant barrier to understanding the evolving digital landscape within the region's unique sociocultural contexts. Projects such as Children and Online Safety in the Pacific aims to identify issues and benefits arising from increased access to technology in the Solomon Islands, Papua New Guinea, Kiribati, and Fiji.

Research is vital to inform evidence-based interventions and policies. Effective ways to help children manage social media necessitates the involvement of everyone, from parents to policy makers. Drawing from the broader literature on digital technology among young people, there is a need for greater education and awareness to promote high-quality digital content and help guide parents on effective strategies to facilitate healthy digital habits early in life. This may be especially beneficial for parents with less formal education, with higher parental education linked to active parental mediation strategies and digital literacy among Chinese adolescents, for example. Parents from Singapore and Australia with greater digital literacy and parental self-efficacy were more inclined to adopt mediation strategies. Furthermore, multifaceted health promotion approaches and policies are essential due to the links between digital technology use and body-mass index and obesity among young people in the region, driven by increased sedentary behaviour, reduced physical activity, and unhealthy eating choices. Importantly, young people must be actively engaged and involved in the decision making process at the core of interventions and policies regarding social media to facilitate healthy digital technology habits and support positive developmental health outcomes in a rapidly evolving digital landscape.

While social media has become an integral aspect of young people's lives, its effects on brain development are inevitably complex and highly influenced by context. Effective guidance, access to high-quality content, active parental involvement, and meaningful engagement with young people are vital for mitigating negative impacts and promoting positive health outcomes. Ultimately, much work needs to be done to understand the potential health consequences of outright bans, including their impact on young people's ability to connect with others. Despite differing opinions, one thing is certain: balancing young people's need for connection with the need to safeguard their health and help them thrive in the digital world is fundamental. We at *The Lancet Regional Health—Western Pacific* stand together with all members of our community to ensure our collective growth and positive health outcomes for all in this new era of advancements in digital technology and artificial intelligence.

